# Supplementary material for: Factors associated with respectful maternity care during hospital deliveries: A cross-sectional study in Bangladesh
Source: PLoS One. 2025 Oct 17;20(10):e0334418. doi: 10.1371/journal.pone.0334418 (PMC12533879; doi:10.1371/journal.pone.0334418)
Supplement: S1 File — (PDF) [file pone.0334418.s001.pdf]

[Questionnaire-English]

Title: Exploring Respectful Maternity Care: A Comparative study on women's experiences in private and public hospitals

Data Collection Form

[Please read the following questions carefully. Encircle the code according to your response.]

ID. No: .... **Date: ....**

1. Sociodemographic characteristics

No	Questions	Responses	Code
**1.1**	**Age (years)**		
**1.2**	**Level of Education**	Unable to read and write	1
		Able to read and write	2
		Primary	3
		Secondary	4
		College and above	5
**1.3**	**Marital status**	Married	1
		Others	2
**1.4**	**Residence**	Urban	1
		Rural	2
**1.5**	**Occupation**	Housewife	1
		Service holder (government/non-government)	2
		Business owner	3
		Others	4

2. Obstetrics related questions

No	Questions	Responses	Code
**2.1**	**Parity**	Primipara	
		Multipara	
**2.2**	**Antenatal care (ANC) follow-up**	Yes	
		No	
**2.3**	**Number of ANC visit**	<4	
		≥4	
**2.4**	**Place of delivery**	Private hospital	
		Public hospital	

**2.5**	**Reason to deliver in hospital**	Planned	
		Referred	
**2.6**	**Length of labor (hours)**	<12	
		≥12	
**2.7**	**Mode of delivery**	Normal vaginal delivery	
		Instrumental assisted	
		Cesarean section	
**2.8**	**Companion during labor**	Yes	
		No	
**2.9**	**Sex of labor attendant**	Male	
		Female	
**2.10**	**Maternal outcome**	Normal	
		With complication	

3. Status of respectful maternity care (RMC)

**Categories**	**Items of RMC**	**Responses**				
		**1-strongly disagree**	**2-disagree**	**3-neutral**	**4-agree**	**5-strongly agree**
**Friendly care**	I felt that healthcare workers cared for me with a kind approach					
	Healthcare workers treated me in a friendly manner					
	The healthcare providers were talking positively about pain and relief					
	The health worker showed his/her concern and empathy					
	All healthcare workers treated me with respect as an individual					
	The healthcare workers speak to me in a language that I can understand					
	The healthcare providers called me by my name					
**Abusive-free care**	The healthcare workers responded to my needs whether or not I asked					

	Some healthcare providers slapped me during delivery for different reasons					
	Some health workers shouted at me because I haven't done what I was told to do					
**Timely-care**	I was kept waiting for a long time before receiving services					
	Service provision was delayed due to the health facilities' internal problem					
**Discrimination-free care**	Some of the health workers do not treat me well because of some personal attribute					
	Some health workers insulted me and my companions due to my personal attributes					
	I was allowed to practice cultural rituals in the facility					

Thank you for your participation.
