# Supplementary material for: Factors associated with respectful maternity care during hospital deliveries: A cross-sectional study in Bangladesh
Source: PLoS One. 2025 Oct 17;20(10):e0334418. doi: 10.1371/journal.pone.0334418 (PMC12533879; doi:10.1371/journal.pone.0334418)
Supplement: Attachment — Submitted filename: 1330-Reviewer correction.docx [file pone.0334418.s005.docx]

**Reviewer #2:**

You answered all reviewers' questions well. I have a few small items, and then this is ready for publication, in my view.

Line 80 refers to Bangladesh as “a developing country of south-east Asian region.” Bangladesh is in South Asia, not Southeast Asia. The phrase “developing country” has been superseded by the specific income level of the country named. In the case of Bangladesh, it is a lower-middle-income country. Please change the phrase on line 80 to “a lower-middle-income country in South Asia.” Please review this World Bank list if needed: https://datahelpdesk.worldbank.org/knowledgebase/articles/906519-world-bank-country-and-lending-groups.

Thank you for the information about education levels. I’m noting that your study includes much higher percentages of women educated to upper secondary and university levels than Bangladesh generally. This likely tracks closely with socioeconomic status, and you mention in your limitations that you do not handle socioeconomic disparities in detail. That is reasonable (because most of the population you are capturing are those who can go to a private hospital) and so no change is needed there. However, you could also include a sentence or two in the discussion/limitation section noting that you captured a cohort that skews more highly educated than the female population of Bangladesh generally, and this limits how generalizable your findings are.

In Table 3, you use the term “cOR.” I assume you mean “crude odds ratio.” Please spell out the full term somewhere in the document.

Finally, Table 3 shows some dramatic shifts between your cOR and aOR values, including some changes in direction (eg, with respect to facilities referrals, from cOR of 0.56 (0.38, 0.83)* to aOR of 2.88 (1.59, 5.31), so changing from lower likelihood to higher likelihood of respectful care). Is this due to small sample sizes? Please review closely and ensure all values are correct, and please comment on these shifts in your discussion or limitation section.

***Authors’ Response:***

*We sincerely thank the reviewer for the careful review of our manuscript and for the insightful and constructive comments.*

*First, we have updated line 80 to “a lower-middle-income country in South Asia” per your suggestion.*

*Second, we acknowledged that our cohort includes a higher proportion of women with upper secondary and university education than the general female population, which may limit generalizability; this has been added to the limitations section of the manuscript.*

*Third, the term “cOR” means “crude odds ratio (cOR)” which is now mentioned in the footnote of Table 3.*

*According to your suggestion, we reviewed the analysis and we agree that shifts between cOR and aOR, including reversals (e.g., facility referrals), likely reflect confounding and the relatively small sample size. This has been noted in the limitations section of the manuscript.*

*Finally, we greatly appreciate the reviewer’s thoughtful guidance in strengthening the manuscript.*

**Reviewer #3:**

Thank you for your revisions. I have reviewed the updated manuscript and find the changes to be satisfactory. I have no additional comments.

***Authors’ Response:***

*We sincerely thank the reviewer for taking the time to review our revised manuscript and for the positive feedback. We greatly appreciate your acknowledgment and support.*
